# The effect of conservation agriculture technologies adoption on food production and security in Northern Malawi: evidence from Mzimba district

**DOI:** 10.3389/fnut.2025.1615990

**Published:** 2025-07-09

**Authors:** George N. Chidimbah Munthali, He Puming, Lazarus Obed Livingstone Banda, Peter Stephen Donald Ngulube, Gama Rivas Daru, Thokozani Mzumara, Moses M. N. Chitete, Zondiwe Mabilabo Jere

**Affiliations:** ^1^School of Economics and Management, Yangtze University, Jingzhou, Hubei, China; ^2^Finance Department, Mzuzu University, Mzuzu, Malawi; ^3^Nalikule College of Education, Lilongwe, Malawi; ^4^Department of Biological Sciences, Malawi University of Science and Technology, Blantyre, Malawi; ^5^School of Marxism, Chang’an University, Xi’an, Shaanxi, China; ^6^Department of Ophthalmology, Mzimba-North District Health Office, M’bwelwa District Council, Mzuzu, Malawi; ^7^Centre for Agricultural Research and Development, Lilongwe University of Agriculture and Natural Resources, Lilongwe, Malawi; ^8^Department of Agri-Sciences, Mzuzu University, Mzuzu, Malawi

**Keywords:** conservation agriculture technologies, rural farmers, SSA, food security, sustainable agriculture

## Abstract

**Background:**

Adoption of conservation agriculture technologies (CATs) has emerged as a strategy to improve farm productivity and achieve food security in many parts of the world.

**Aim:**

This study aimed to assess the effect of CAT adoption on food production and security.

**Methods:**

This quantitative cross-sectional study was conducted in the Vibangalala Extension Planning Area (EPA) in Mzimba district, Northern Malawi. Data was collected using structured questionnaires. The data were entered in SPSS version 27. Descriptive statistics included frequency, percentage, weighted percentage, means, standard deviations, and standard error mean. Inferential statistics included a *t*-test and a linear regression model, with the value of *p* < 0.05 considered statistically significant.

**Findings:**

The study found that households that adopted CATs have high food production and experience food security compared to non-adopters. Adoption of CATs significantly boosts production, with adopters experiencing lower food insecurity and better dietary diversity than non-adopters.

**Conclusion and recommendation:**

The study confirms that adopting conservation agricultural technologies is integral to improving agricultural production, but remains entangled in disparities related to gender, farm size, and education. Efforts should be made to ensure equity and equality in conservation agricultural technology adoption to improve food security for all.

## 1 Introduction

Food insecurity remains the main challenge for many developing countries, particularly in Sub-Saharan Africa (SSA), where over 60% of the population depends on agriculture for their livelihoods (1, 2). Despite global efforts to address this issue, the region continues to face persistent challenges related to low agricultural productivity, climate variability, and environmental degradation, which threaten food security across the continent.

Within SSA, agriculture is predominantly characterized by smallholder farmers whose livelihoods depend primarily on rainfed farming. Low adoption of conservation agricultural technologies across many SSA countries hampers food production, yet many scholars recognize technological adoption as a critical pathway for eradicating food insecurity, reducing poverty, and improving living standards (3). Rogers (4) defines technology adoption as a decision to fully utilize an innovation as the best course of action available. Technologies such as improved seeds, fertilizers, irrigation, and conservation agriculture have been widely adopted globally to boost yields and reduce harvest losses (5).

In this context, practices like mulching, intercropping, crop rotation, and the use of crop residues on the soil surface are considered environmentally beneficial strategies. These practices are recommended for smallholder farmers as safe and sustainable responses in this era of climate change, particularly in semi-arid regions (6–8).

A recent study examining the effect of conservation agriculture (CA) adoption on household food security among smallholder maize farmers in Ghana found that CA practices significantly enhance food security, influenced by socio-economic factors such as farmer age, gender, education, household size, and access to extension services (9). Another by Wagstaff and Harty (10) examined the impact of conservation agriculture (CA) on food security in three low veldt districts of Zimbabwe. Their evaluation of Concern Worldwide’s CA program demonstrated that, even in semi-arid regions characterized by environmental challenges, the adoption of CA practices could positively influence household food security by improving crop yields and enhancing dietary diversity. This evidence underscores the potential of CA as a resilient and sustainable farming system in similar vulnerable environments, emphasizing its relevance for regions facing climate variability and resource constraints (10).

Focusing specifically on Malawi, despite efforts by the government and development partners such as the International Fund for Agricultural Development (IFAD), Food and Agriculture Organization (FAO), and Civil Society Agriculture Network (CISANET)—food insecurity remains high, affecting nearly 50% of the population who live below the poverty line and face recurrent shortages (11). The country’s low agricultural productivity results from multiple challenges, including environmental degradation, limited access to farm inputs, low mechanization, poor market linkages, and restricted irrigation options (12). To sustainably feed its growing population, Malawi must increase agricultural productivity amidst climate shocks, small landholdings, and rising land degradation levels (13). These factors hinder smallholder farmers’ ability to produce stable food supplies (12, 14).

Several studies provide strong evidence that the adoption of specific agricultural technologies can significantly enhance food production and household food security. For example, the use of fertilizer trees has been shown to improve soil fertility and maize yields in six districts in Malawi (15). Similarly, sustainable intensification practices such as maize-legume intercropping, crop rotation, and minimum tillage have led to improved food security outcomes at the household level (16, 17). In addition, recent innovations such as drone technology have enhanced farm knowledge and decision-making, enabling farmers to increase productivity with fewer inputs (18). Improved bean varieties have also demonstrated benefits, including higher grain yield and reduced losses under environmental stress (19). The introduction of the Farm Input Subsidy Program (FISP) has significantly increased national cereal production, especially maize (11). Prior to FISP, the Starter Pack Program (SPP), launched in 1998, aimed to improve access to improved seeds, legumes, and fertilizers for smallholder farmers, although its impact was limited (12). These interventions reflect broader efforts to promote technology adoption as a means to boost productivity.

Despite these challenges, increasing and sustaining agricultural productivity remains essential for achieving food security in Malawi (20). Promoting the adoption of sustainable farming practices like conservation agriculture (CA) appears vital, especially considering the impacts of climate change. According to Kassam et al. (21), CA is “a farming system that promotes maintenance of a permanent soil cover, minimum soil disturbance, and diversification of plant species.” It enhances biodiversity and biological processes both above and below ground, leading to better water and nutrient efficiency and improved crop yields (21). CA principles include minimal soil disturbance, permanent soil cover, and crop rotation, with technologies such as agroforestry, intercropping, box ridging, manure application, permanent planting basins, and swales (22). Despite these promising findings, the benefits of technology adoption are not uniform across regions or farming systems. Factors such as soil quality, access to markets, and availability of extension services significantly influence outcomes. Farmers with better access to extension services report higher yields due to improved understanding and implementation of technologies (23). Moreover, participation in season-long extension programs leads to greater adoption compared to short-term training events (24). However, the adoption of CA technologies in Malawi among smallholder farmers remains limited, mainly due to limited credit facilities, inadequate extension services, and low education levels, especially in rural areas where subsistence farming dominates (25). A study by Jew et al. (26) examining the constraints faced by smallholder farmers in Malawi during climate shocks highlights the potential of conservation agriculture (CA) as a resilient and adaptable solution. The research underscores how systemic issues such as household health, access to inputs, labor shortages, and institutional support significantly influence the adoption of CA and overall agricultural productivity. Despite its agronomic benefits, adoption remains low due to socio-economic, structural, and institutional barriers, emphasizing the need for holistic approaches that address these underlying challenges alongside technological promotion (26).

In Malawi CA is one of the major agricultural development policies; however, adoption of CA remains as low as 10 percent (27). Previous efforts to improve adoption of CA in Malawi and other African settings have remained futile (28). While previous studies have examined the impact of conservation agriculture technologies (CATs) on food security at regional or national scales [e.g., (26, 29)], this study provides localized evidence from Vibangalala EPA in Mzimba district, Northern Malawi a historically prioritized area for CAT promotion. The current research contributes by exploring how socio-economic disparities, farm size, and environmental conditions shape the relationship between technology adoption and food security outcomes. The study aimed to bridge the gap in understanding the relationship between conservation agricultural technologies adoption and its effects on food production and household food security among smallholder farmers in the Vibangalala Extension Planning Area (EPA), Mzimba district. Specifically, it addresses the lack of detailed knowledge on how the level of technology adoption influences food output and household wellbeing, as well as the factors that drive adoption. This fills an important gap by clarifying the connections and causality among these variables within this particular context.

This study is part of the broader research project introduced in Chidimbah Munthali et al. (30), where the first objective was focused on documenting and analyzing the diversities of conservation agriculture technologies (CATs) adopted by smallholder farmers in Vibangalala EPA. The current work extends this effort by focusing on effects of CAT adoption on food production and household food security, building upon the findings previously reported.

## 2 Materials and methods

### 2.1 Study design

The study employed a quantitative cross-sectional design that involved collecting data from households in the Vibangalala EPA), Mzimba district. The EPA is the lowest administrative unit for planning and implementation of agricultural programs in a district (31). The goal was to measure the relationship between challenges and opportunities in adopting conservation agricultural technologies through the observation and use of questionnaires to collect data.

### 2.2 Study setting

**FIGURE 1:**
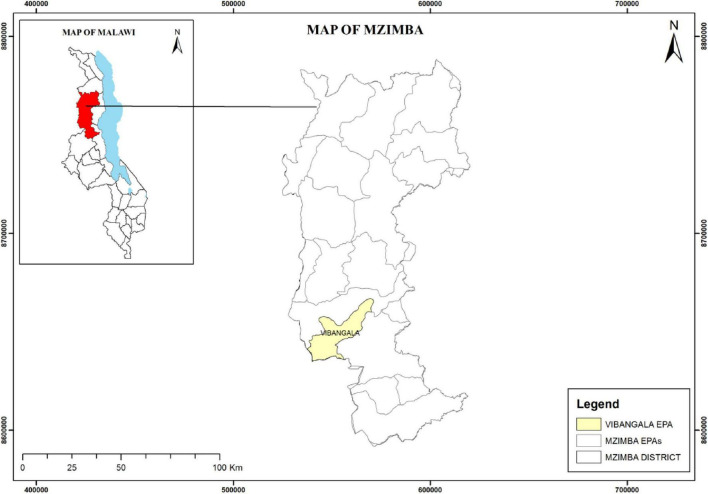
Map of Vibangalala in Mzimba district, Malawi. Source: Authors, 2025 (using ArcGIS 4).

The study was conducted in Vibangalala EPA, Mzimba district, in Northern Malawi. The district covers an area of 10,430 km^2^ and has a population of 610,944 and 47,060 households (32). The study area was purposively selected because it was one of the first areas where promotion of CATs was conducted. The area receives approximately 159.08 mm of rain annually. Most of the households around Vibangalala EPA adopt conservation agriculture technologies to mitigate climate challenges and achieve financial sustainability. A map of the study site of Vibangalala EPA is shown below (Figure 1).

### 2.3 Population and sampling

Vibangalala EPA had a total population of 13,152. To come up with a replacement sample size, the researchers used the following formula (Equation i).


$$\mathrm{sample}\,\mathrm{size}\,\left(\mathrm{SS}\right)=\frac{z^{2*p\,\left(1-p\right)}}{e^{2}}\,\mathrm{Equationi}$$

With the given Vibangalala area, we adjusted the sample using a second formula (Equation ii) witha 95% level of confidence. We found the z level to be within 1.96.


$$\mathrm{Adjusted}\,\mathrm{sample}\,\mathrm{size}=\frac{\mathrm{SS}}{1+\frac{\left(\mathrm{SS}-1\right)}{N}}\,\mathrm{Equationii}$$

From equation (i) we will have $S\,S=\frac{1.96^{2}*0.5\,\left(1-0.5\right)}{0.05^{2}}$ 384.16

Substituting SS into equation (ii), we have $\frac{384.16}{1+\frac{\left(384.16-1\right)}{13152}}\,373.285$

Furthermore, we found out that the sample size without the ± 5% precision error is 373. And then, we included a positive 5% of the total sample size, 373, which gives us 18.65, ≅ 19 more samples. This totaled a sample size of 392, and we chose to use 390 respondents as it falls between the minimum and maximum values.

Based on statistical power and validity calculations, the study employed a sample size of 390 participants. The participants were selected using a stratified sampling technique, and within each stratum, participants were randomly selected to ensure equal representation of the study population.

### 2.4 Data collection

In this study, data were collected by trained research assistants who were familiar with the aim and objectives of the study as well as the data collection tool. The team was led by Agricultural Extension Development Officers (AEDOs) who were responsible for the study area and were quite knowledgeable about the study area. The research instrument was pre-tested through a pilot study, and changes were made to improve the instrument. The research instrument for this study was a structured questionnaire that contained closed-ended questions on gender, age group, marital status, level of education, occupation, type of contract, household size, occupation, type of land ownership, and size of farm.

### 2.5 Data analysis

The data was entered in Statistical Package for Social Sciences (SPSS) Version 27 (25). Descriptive statistics included frequencies, percentages, weighted percentages, mean, standard deviation, and standard error mean. To illustrate the data graphically, the study employed graphs and tables. For inferential statistics, the study employed an independent *t-*test to assess mean differences between variables. The value of *p* < 0.05 was considered statistically significant.

### 2.6 Model construction

The multiple linear regression model used in this context is expressed as:


Y⁢b⁢0+(b⁢1⁢x⁢1)+(b⁢2⁢x⁢2)+…+(b⁢9⁢x⁢9)+ε

Where:

Y is the observed value of the dependent variable,

b0 is the intercept of the model, b1,b2,…,b9 are the coefficients corresponding to each explanatory variable, x1,x2,…,x9 are the independent variables influencing food production, εε represents the error term.

In this model, b0 is the expected value of Y when all independent variables are zero. The coefficients b1 through b9 quantify the effect of each variable, such as food security level, marital status, occupation, distance to market, conservation of conservation agricultural technologies, soil type, temperature, and soil quality, on food production.

## 3 Results

Significant differences between those who adopt and those who do not adopt conservation agricultural technologies in Malawi are uncovered by the descriptive analysis in Table 1. Gender plays a pivotal role, as the majority of adopters (58%) are male farm managers. In comparison, only 39.1% of non-adopters hold this role, indicating potential gender-based disparities in resource access and decision-making processes. Farm size also proves to be a crucial factor, as the average adopter manages 1.68 hectares while the average non-adopter manages only 1.04 hectares, showcasing how resource availability impacts adoption rates. Adopters whose households tend to be larger may benefit from the contribution of family labor in adopting new practices. Adopters also exhibited higher levels of education and exposure to conservation agriculture technologies (CAT) training, underscoring the crucial role of disseminating knowledge and increasing capacity in fostering adoption. Furthermore, adopters reported better access to extension services and improved environmental conditions due to slight improvements in soil quality, potentially enhancing the practicality and advantages of implementing these technologies. The results highlight the dynamic relationship among socio-economic, institutional, and environmental elements in influencing the implementation of agricultural advancements, offering valuable perspectives on methods for fostering sustainable food cultivation and stability in Malawi.

**TABLE 1:** Descriptive statistics of variables used in the regression.

Explanatory variables	Category	Adopters	Non-adopters
Gender of the farm manager	Male	213 (58%)	9 (39.1%)
	Female	154 (42%)	14 (60.1%)
Age group	Mean, SE	2.39, 0.76	2.43, 0.84
Education level	Mean, SE	1.18, 0.42	1.13, 0.34
Size of farm	Mean, SE	1.68, 0.76	1.04, 0.21
Marital status	Mean, SE	0.92, 0.27	0.83, 0.39
Occupation	Mean, SE	1.10, 0.46	1.00, 0.00
Household size	Mean, SE	2.65, 0.53	2.57, 0.73
Distance to nearest market (km)	Mean, SE	2.85, 0.37	2.96, 0.21
Type of land ownership	Mean, SE	1.02, 0.13	1.00, 0.00
Type of farming	Mean, SE	1.01, 0.12	1.00, 0.00
Heard about CATs	Mean, SE	1.00, 0.05	1.48, 0.51
Extension access	Mean, SE	1.01, 0.10	1.04, 0.21
Farming experience	Mean, SE	1.52, 0.50	1.57, 0.51
Ownership of smartphone and radio	Mean, SE	1.39, 0.49	1.43, 0.51
Belonging to a club	Mean, SE	1.22, 0.41	1.48, 0.51
Attendance to CATs training	Mean, SE	1.17, 0.38	1.04, 0.21
Type of soil	Mean, SE	1.10, 0.32	1.04, 0.21
Temperature	Mean, SE	1.02, 0.13	1.09, 0.29
Rainfall	Mean, SE	2.06, 0.24	2.00, 0.00
Soil quality	Mean, SE	1.01, 0.12	1.00, 0.00

The negative occupation coefficient (−0.022) at a significance of 5% suggests the likelihood of non-farming occupations reducing food production Table 2. The level of food security (0.565, 1%) positively impacts food production; families who have food are likely to venture into more food production, unlike families who are deprived of food. Environmental variables, such as temperature (−0.099, 1%), soil quality (−0.187, 1%), and the type of soil (−0.043, 1%), indicate that higher temperatures, poor soil quality, and sandy soil type reduce food production. Social factors like marital status (0.036, 5%) and distance to markets (0.034, 5%) highlight the role played by social stability and proximity to economic hubs in improving food productivity; married farmers are more likely to engage in farming activities, unlike single farmers. Moreover, the complexity of working with CATs (0.196, 1%), more so on a moderate level, promotes food production.

**TABLE 2:** Multiple linear regression on factors influencing food production.

Variables	Coef.	SE
Income after adoption of technologies	-0.001	0.006
Age group	0.007	0.006
Education level	-0.009	0.011
Size of farm	-0.006	0.007
Impact of adoption on food security	0.565***	0.034
Gender of the farm manager	-0.001	0.009
Marital status	0.036**	0.017
Occupation	-0.022**	0.011
Household size	0.000	0.008
Distance to nearest market (km)	0.034**	0.014
Type of land ownership	0.014	0.038
Type of farming	0.035	0.038
Heard about CATs	0.011	0.025
The gender that makes the most decisions on farming	-0.001	0.006
Rating on time	-0.005	0.022
Complexity of working with CATs	0.196***	0.033
Extension access	0.015	0.039
Farming experience	0.003	0.009
Ownership of smartphone & radio	0.003	0.011
Belonging to a club	0.000	0.013
CAT training	0.010	0.016
Type of soil	-0.043***	0.015
Temperature	-0.099***	0.030
Rainfall	0.018	0.019
Soil quality	-0.187***	0.040
Adopted CAT because of economic stability	-0.006	0.004
Adopted CAT because of social influence	-0.003	0.004
Adopted CAT because of environmental factors	0.000	0.004

** and *** indicate significance at the 10%, 5%, and 1% levels, respectively. Coef. is the coefficient; SE is the standard error.

From Table 3, households that adopted technologies experienced lower food insecurity frequencies as compared to non-adopters. About 94.4% of adopters indicated having rarely had a limited variety of foods, while non-adopters had slightly higher percentages in the “sometimes” (6.9%) and “rarely” (5.6%) categories, suggesting adopters have better dietary variety. Similarly, 94.4% of adopters rarely ate undesired foods, compared to 5.6% of non-adopters. Additionally, 95.6% of adopters reported rarely eating smaller meals than needed. On the other hand, 93.6% of adopters are rarely worried about having enough food, while 91.4% are sometimes worried, showing that adopters are slightly less anxious about food supplies.

**TABLE 3:** Descriptive food security frequencies.

Variables	Have you adopted CAT		Total
	Yes	No	
**Did you or any household member have to eat a limited variety of foods?**			
1.00 rarely (once or twice)	168	10	178
	94.4%	5.6%	100.0%
2.00 sometimes (3–10 times)	148	11	159
	93.1%	6.9%	100.0%
3.00 often (more than 10 times)	51	2	53
	96.2%	3.8%	100.0%
**Did you or any household member have to eat some foods that you really did not want to eat**			
1.00 rarely (once or twice)	167	10	177
	94.4%	5.6%	100.0%
2.00 sometimes (3–10 times)	146	10	156
	93.6%	6.4%	100.0%
3.00 often (more than 10 times)	54	3	57
	94.7%	5.3%	100.0%
**Did you or any household member have to eat a smaller meal than you felt you needed**			
1.00 rarely (once or twice)	151	7	158
	95.6%	4.4%	100.0%
2.00 sometimes (3–10 times)	154	13	167
	92.2%	7.8%	100.0%
3.00 often (more than 10 times)	62	3	65
	95.4%	4.6%	100.0%
	Have you adopted a technology		Total
	1.00 yes	2.00 no	
**Did you worry that your household would not have enough food?**			
1.00 rarely (once or twice)	132	9	141
	93.6%	6.4%	100.0%
2.00 Sometimes (3–10 times)	128	12	140
	91.4%	8.6%	100.0%
3.00 Often (more than 10 times)	107	2	109
	98.2%	1.8%	100.0%
**Were you or any household member not able to eat the kinds of foods you preferred?**			
1.00 rarely (once or twice)	171	14	185
	92.4%	7.6%	100.0%
2.00 sometimes (three to ten times)	144	8	152
	94.7%	5.3%	100.0%
3.00 often (more than ten times)	52	1	53
	98.1%	1.9%	100.0%
	Have you adopted a technology?		Total
	1.00 yes	2.00 no	
**Did you or any household member have to eat fewer meals in a day?**			
1.00 rarely (once or twice)	147	8	155
	94.8%	5.2%	100.0%
2.00 sometimes (3–10 times)	161	13	174
	92.5%	7.5%	100.0%
3.00 often (more than 10 times)	59	2	61
	96.7%	3.3%	100.0%
**Was there ever no food to eat of any kind**			
1.00 rarely (once or twice)	140	7	147
	95.2%	4.8%	100.0%
2.00 sometimes (3–10 times)	172	14	186
	92.5%	7.5%	100.0%
3.00 often (more than 10 times)	55	2	57
	96.5%	3.5%	100.0%
**Did you or any household member go to sleep at night hungry?**			
1.00 rarely (once or twice)	144	9	153
	94.1%	5.9%	100.0%
2.00 sometimes (3–10 times)	159	13	172
	92.4%	7.6%	100.0%
3.00 often (more than 10 times)	64	1	65
	98.5%	1.5%	100.0%
**Did you or any household member go a whole day and night without eating?**			
1.00 rarely (once or twice)	144	10	154
	93.5%	6.5%	100.0%
2.00 sometimes (3–10 times)	158	11	169
	93.5%	6.5%	100.0%
3.00 often (more than 10 times)	65	2	67
	97.0%	3.0%	100.0%

The results show that a high proportion of households who “sometimes” and even “often” experienced food insecurity still adopted Climate-smart Agricultural Technologies (CAT). Among those who “often” had to eat a limited variety of foods, 96.2% adopted CAT, and among those who “often” went to sleep hungry, 98.5% were adopters. This trend is consistent across several indicators, suggesting that CAT adopters are not exclusively in the “rarely” food-insecure group. This pattern implies that CAT adoption is not only a result of being food secure but also a response to food insecurity. Households facing frequent food stress (“sometimes” and “often”) may be more motivated to adopt CAT as a coping or resilience strategy to improve food availability and reduce vulnerability. In this context, these groups are “unique” because, despite their exposure to food insecurity, they are still proactive in seeking out adaptive technologies—perhaps even more so than food-secure households. Therefore, these households are not categorized as “rarely” food insecure because they have faced chronic or recurring food access challenges, which might stem from factors such as climate variability, land degradation, or poverty. Their adoption of CAT reflects a need-driven decision rather than a capacity-driven one, underscoring how food-insecure households are not passive victims but active agents in improving their food systems.

The percentages for adopters are generally higher for rare cases across the variables. These results consistently indicate that most households adopting CATs experience food security challenges compared to non-adopters.

To check the significance of each of the household characteristics in Table 2 to food security, the ratings were summed up for each respondent, and the frequencies were determined as shown in Table 4. Since the rates were in three categories, they were multiplied by the nine questions to find a maximum of 27, with the minimum standing at nine. Nine indicates the highest level of food security, while 27 indicates food insecurity among adopters and non-adopters. From Table 4, a good percentage of the adopters fell in the list rating (73, 19.9%), showing that food insecurity was not a problem after adopting the techniques. To find out the factors that influence a better rating, an ordinal regression has been carried out, and the results are recorded in Table 5.

**TABLE 4:** Food security ratings between adopters and non-adopters.

Security ratings	Have you adopted a technology		Total
	Adopters	Non-adopters	
9.00	73	4	77
	19.9%	17.4%	19.7%
10.00	15	1	16
	4.1%	4.3%	4.1%
11.00	6	0	6
	1.6%	0.0%	1.5%
12.00	7	0	7
	1.9%	0.0%	1.8%
13.00	9	2	11
	2.5%	8.7%	2.8%
14.00	14	2	16
	3.8%	8.7%	4.1%
15.00	34	2	36
	9.3%	8.7%	9.2%
16.00	38	4	42
	10.4%	17.4%	10.8%
17.00	43	3	46
	11.7%	13.0%	11.8%
18.00	56	3	59
	15.3%	13.0%	15.1%
19.00	14	0	14
	3.8%	0.0%	3.6%
20.00	12	0	12
	3.3%	0.0%	3.1%
21.00	8	0	8
	2.2%	0.0%	2.1%
22.00	1	1	2
	0.3%	4.3%	0.5%
24.00	3	0	3
	0.8%	0.0%	0.8%
25.00	1	0	1
	0.3%	0.0%	0.3%
27.00	33	1	34
	9.0%	4.3%	8.7%

**TABLE 5:** Ordinal logit regression for factors influencing various food security ratings.

Variables	Coef	SD	Wald	Sig.	95% confidence interval	
					**Lower bound**	**Upper bound**
[(Income before adoption = 1)]	0.118	0.373	0.099	0.753	−0.614	0.849
[(Income before adoption = 2)]	0^a^	–	–	–	–	–
[(Income after adoption = 1)]	−1.899	0.337	31.694	00***	−2.561	−1.238
[(Income after adoption = 2)]	−1.067	0.343	9.659	02***	−1.741	−0.394
[(Income after adoption = 3)]	0^a^	–	–	–	–	–
[(Age = 1)]	0.548	0.285	3.687	0.055*	−0.011	1.107
[(Age = 2)]	−0.136	0.248	0.299	0.584	−0.622	0.350
[(Age = 3)]	0^a^	–	–	–	–	–
[(Education = 1)]	0.475	1.611	0.087	0.768	−2.684	3.633
[(Education = 2)]	0.651	1.637	0.158	0.691	−2.557	3.858
[(Education = 3)]	0^a^	–	–	–	–	–
[(Acre = 1)]	0.571	0.329	39	0.083*	−0.074	1.217
[(Acre = 2)]	0.051	0.310	0.027	0.869	−0.557	0.659
[(Acre = 3)]	0^a^	–	–	–	–	–
[(Food production = 1)]	−3.771	1.713	4.844	0.028**	−7.129	−0.413
[(Food production = 2)]	0^a^	–	–	–	–	–
[(Food Security = 1)]	4.639	1.602	8.388	04***	1.500	7.779
[(Food Security = 2)]	0^a^	–	–	–	–	–
[(Gender = 0)]	−0.092	0.238	0.150	0.698	−0.560	0.375
[(Gender = 1)]	0^a^	–	–	–	–	–
[(Marital Status = 0)]	−0.259	0.422	0.377	0.539	−1.086	0.568
[(Marital Status = 1)]	0^a^	–	–	–	–	–
[(Occupation = 1)]	20.681	2.421	72.980	0.000***	15.936	25.426
[(Occupation = 2)]	21.027	2.498	70.856	0.000***	16.131	25.923
[(Occupation = 3)]	21.071	2.490	71.591	0.000***	16.190	25.952
[(Occupation = 4)]	18.181	00	–	–	18.181	18.181
[(Occupation = 5)]	0^a^	–	–	–	–	–
[(Household Size = 1)]	−0.805	0.625	1.655	0.198	−2.030	0.421
[(Household Size = 2)]	−0.016	0.233	05	0.945	−0.473	0.441
[(Household Size = 3)]	0^a^	–	–	–	–	–
[(Distance = 1)]	17.218	5.484	9.859	0.002***	6.470	27.965
[(Distance = 2)]	3.502	0.415	71.255	0.000***	2.689	4.315
[(Distance = 3)]	0^a^	–	–	–	–	–
[(Land Ownership = 1)]	−0.188	0.992	0.036	0.850	−2.132	1.757
[(Land Ownership = 2)]	0^a^	–	–	–	–	–
[(Type of farming = 1)]	1.480	0.992	2.228	0.136	−0.463	3.423
[(Type of farming = 2)]	0^a^	–	–	–	–	–
[(cat knowledge = 1)]	0.837	0.751	1.244	0.265	−0.634	2.309
[(cat knowledge = 2)]	0^a^	–	–	–	–	–
[(Adopted = 1)]	0.015	0.551	01	0.978	−1.064	1.094
[(Adopted = 2)]	0^a^	–	–	–	–	–
[(Decision makers = 1)]	−0.182	0.280	0.420	0.517	−0.731	0.368
[(Decision makers = 2)]	1.051	0.336	9.774	0.002***	0.392	1.710
[(Decision makers = 3)]	0^a^	–	–	–	–	–
[(Time rating = 1)]	0.810	1.686	0.231	0.631	−2.495	4.115
[(Time rating = 2)]	0.388	1.832	0.045	0.832	−3.204	3.980
[(Time rating = 3)]	0^a^	–	–	–	–	–
[(Complexity = 1)]	4.941	2.184	5.116	0.024**	0.659	9.222
[(Complexity = 2)]	0^a^	–	–	–	–	–
[(Complexity = 3)]	0^a^	–	–	–	–	–
[(Extension access = 1)]	−0.755	1.018	0.551	0.458	−2.750	1.240
[(Extension access = 2)]	0^a^	–	–	–	–	–
[(Farming experience = 1)]	−0.542	0.211	6.617	0.010***	−0.954	−0.129
[(Farming experience = 2)]	0^a^	–	–	–	–	–
[(Ownership smartphone = 1)]	−0.153	0.278	0.302	0.583	−0.697	0.392
[(Ownership smartphone = 2)]	0^a^	–	–	–	–	–
[(Belonging club = 1)]	−0.250	0.313	0.634	0.426	−0.864	0.365
[(Belonging club = 2)]	0^a^	–	–	–	–	–
[(CAT Training = 1)]	−1.234	0.412	8.975	0.003***	−2.041	−0.427
[(CAT Training = 2)]	0^a^	–	–	–	–	–
[(Type soil = 1)]	0.327	1.834	0.032	0.859	−3.267	3.920
[(Type soil = 2)]	0.167	1.866	08	0.928	−3.490	3.825
[(Type soil = 3)]	0^a^	–	–	–	–	–
[(Temperature = 1)]	1.386	0.844	2.697	0.101	−0.268	3.041
[(Temperature = 2)]	0^a^	–	–	–	–	–
[(Rainfall = 1)]	4.655	2.065	5.084	0.024**	0.609	8.701
[(Rainfall = 2)]	1.067	0.529	4.060	0.044**	0.029	2.105
[(Rainfall = 3)]	0^a^	–	–	–	–	–
[(Soil quality = 1)]	−0.759	1.010	0.564	0.453	−2.739	1.222
[(Soil quality = 2)]	0^a^	–	–	–	–	–
[(Economic stability = 1)]	0.083	0.493	0.028	0.867	−0.884	1.049
[(Economic stability = 2)]	−1.022	0.661	2.389	0.122	−2.318	0.274
[(Economic stability = 3)]	−0.230	0.596	0.148	0.700	−1.398	0.939
[(Economic stability = 4)]	0.080	0.522	0.023	0.878	−0.943	1.103
[(Economic stability = 5)]	0^a^	–	–	–	–	–
[(Social influence = 1)]	0.286	0.412	0.482	0.488	−0.522	1.094
[(Social influence = 2)]	0.749	0.417	3.230	0.072*	−0.068	1.566
[(Social influence = 3)]	0.328	0.435	0.568	0.451	−0.525	1.182
[(Social influence = 4)]	0.212	0.476	0.198	0.656	−0.721	1.144
[(Social influence = 5)]	0^a^	–	–	–	–	–
[(Environmental = 1)]	0.609	0.451	1.826	0.177	−0.274	1.493
[(Environmental = 2)]	0.486	0.480	1.026	0.311	−0.454	1.426
[(Environmental = 3)]	0.051	0.460	0.012	0.912	−0.852	0.953
[(Environmental = 4)]	−0.053	0.522	0.010	0.920	−1.075	0.970
[(Environmental = 5)]	0^a^	–	–	–	–	–
**Model**			**−2 Log Likelihood**	**Chi-square**	**df**	**Sig.**
Intercept only			1876.642	–	–	–
Final			1570.550	306.092	53	0.000
**Goodness-of-fit**						
Pearson			–	7979.542	6043	0.000
Deviance			–	1563.383	6043	1.000
**Pseudo R-square**						
Cox and snell			0.544	–	–	–
Nagelkerke			0.548	–	–	–
McFadden			0.162	–	–	–

0^a^ reference variable. *, ** and *** indicate significance at the 10 %, 5 %, and 1 % levels, respectively. And Coef is abbreviation of coefficient, SD is standard deviation, I United States Dollar (USD): 1,751 Malawi Kwacha (MWK).

Table 5 presents the results of ordinal Logistic regression. The ordinal logistic regression model results indicate a good model fit and significant overall explanatory power. The −2 Log Likelihood value for the final model (1570.550) is substantially lower than that of the intercept-only model (1876.642), and the Chi-square test for model improvement is statistically significant (Chi-square = 306.092, df = 53, *p* < 01), suggesting that the predictors included in the model significantly improve its fit over the null model. The pseudo-R-square values further support this conclusion: Cox and Snell R^2^ = 0.544, Nagelkerke R^2^ = 0.548, and McFadden R^2^ = 0.162. These values indicate that the model explains a moderate proportion of the variance in the dependent variable, with Nagelkerke R^2^ suggesting that about 55% of the variation in the ordinal outcome is explained by the predictors. Regarding goodness-of-fit, the Deviance statistic (1563.383) has a high *p*-value (10), indicating no significant difference between the observed and predicted values and thus a good fit of the model. However, the Pearson statistic is significant (*p* < 01), which may suggest some discrepancies in model fit under certain assumptions, although this test can be overly sensitive in large samples.

Furthermore, an endogeneity test was carried out, and no endogeneity was found in the variables. The robustness test was carried out by removing insignificant covariates, and the results are as recorded in Table 6. From the test, income after adoption, age group, size of the farm, distance to the nearest market, farming decision-makers, farming experience, CAT training, and rainfall were found to be significantly affecting food security.

**TABLE 6:** Robustness test.

Dependent variable	Total food security					
**Parameter**	**Coef**	**Robust SD^a^**	**t**	**Sig.**	**95% confidence interval**	
					**Lower bound**	**Upper bound**
Intercept	−2.101	27.429	−0.077	0.939	−56.056	51.854
[(Income after adoption = 1)]	−3.750	0.582	−6.439	00***	−4.896	−2.604
[(Income after adoption = 2)]	−2.260	0.694	−3.256	01***	−3.625	−0.894
[(Income after adoption = 3)]	0^b^	–	–	–	–	–
[(Age = 1)]	0.996	0.546	1.823	0.069***	−0.078	2.070
[(Age = 2)]	−0.288	0.529	−0.544	0.587	−1.329	0.753
[(Age = 3)]	0^b^	–	–	–	–	–
[(Acre = 1)]	1.255	0.735	1.706	0.089*	−0.192	2.701
[(Acre = 2)]	0.266	0.668	0.399	0.690	−1.047	1.580
[(Acre = 3)]	0^b^	–	–	–	–	–
[(Distance = 1)]	29.332	29.151	16	0.315	−28.010	86.674
[(Distance = 2)]	7.040	0.891	7.902	00***	5.288	8.793
[(Distance = 3)]	0^b^	–	–	–	–	–
[(Decision makers = 1)]	−0.215	0.602	−0.357	0.721	−1.400	0.969
[(Decision makers = 2)]	2.036	0.674	3.020	03***	0.710	3.362
[(Decision makers = 3)]	0^b^	–	–	–	–	–
[(Farming experience = 1)]	−13	0.422	−2.376	0.018**	−1.833	−0.173
[(Farming experience = 2)]	0^b^	–	–	–	–	–
[(CAT training = 1)]	−2.021	0.864	−2.340	0.020**	−3.720	−0.322
[(CAT training = 2)]	0^b^	–	–	–	–	–
[(Rainfall = 1)]	7.702	8.790	0.876	0.382	−9.589	24.992
[(Rainfall = 2)]	2.057	0.871	2.362	0.019**	0.344	3.770
[(Rainfall = 3)]	0^b^	–	–	–	–	–

0^b^ reference variable; ** and *** indicate significance at the 10%, 5%, and 1% levels, respectively. And Coef is abbreviation of coefficient; SD is standard deviation.

As shown in Table 5, income after adoption of below 28 USD and 28–57 USD shows a negative and statistically significant (−1.899, −1.067), meaning that low income even after adoption of a technique is likely to lead to more food insecurity, while higher income of more than 57 USD is likely to lead to stable food security. Being in the age group of 18–25 years positively influences food security (0.548). Youths aged 18–25 are more energetic, and when they participate in farming, food insecurity levels reduce. A farm size of less than one acre also increases food security levels (0.571); this is because of the ease of applying CAT in smaller farms than in larger farms. A moderate food production level affects food security negatively (−3.771); this would mean that experiencing highly impactful food production leads to stronger food security. Having an occupation in crop farming, livestock keeping, and trading highly impacts food security, unlike being in piecework and formal employment. Being in the said occupation increases food security by 20.681, 21.027, and 21.071 times, respectively. Similarly, being less than 1 km away from the nearest marketplace and a distance between 1 and 2 km promotes food security by 17.218 and 3.502 times, respectively; having a marketplace 2 km or more away increases food insecurity. Moreover, adopting CATs that are less complicated promotes food security 4.941 times more than the complex CATs. Gender of decision-maker, farming experience, CATs training, rainfall, and social influence also promote food security at [1.051, −0.542, −1.234, (4.655, 1.067), 0.749], respectively.

## 4 Discussion

Agricultural performance, together with food safety, benefits from the effects of implementing agricultural technology methods, including crop rotation, intercropping, and mulching alongside no-tillage systems. The study results show that implementing these conservation agriculture technologies generates sustainability issues that need detailed evaluation, especially regarding economic sustainability, environmental sustainability, and social sustainability. This paper examines the relationship between agricultural technology benefits and challenges and their impact on raising levels of awareness and informed adoption decisions based on sustainability findings from scholarly works.

### 4.1 Impact of agricultural technology on food production and security

Even though improved food production stands as an essential outcome, it does not benefit all households that adopt these technologies. The implementation of technology depends on various variables consisting including farm dimensions together with ground quality, combined climatic conditions, as well as sales channels. The effectiveness of larger farms in producing better results follows the established literature about resource availability, particularly land and capital, affecting the adoption of complex technologies (23, 33). The research discovered that conservation techniques such as intercropping and no-tillage bring higher benefits to farmers who operate smaller farms with less than 1 acre of land (8). The positive trend identifies implementation obstacles for small-scale farms to effectively scale up their technology usage, thus requiring specialized support to enhance performance. These findings are supported by research conducted in regions like Mangwe district, Zimbabwe, where conservation agriculture practices such as intercropping, no-tillage, and mulching have demonstrated potential to improve food security and livelihoods among smallholder farmers (34). The study highlights that the effectiveness of such practices relies on contextual adaptation, addressing labor demands, climatic conditions, and resource access. Policy support and targeted training are crucial to overcoming adoption barriers and ensuring sustainable benefits.

### 4.2 Socio-economic factors influencing adoption and food security

The implementation of agricultural technology depends heavily on social and economic factors for both good and poor results. Research data supports the fact that men who work in farming adopt new technologies more often than women (35). The food insecurity affecting female-headed households intensifies because women face restricted access to land, capital, and education, which prevents them from adopting new farming technologies (35). The adoption rates reveal that education level, combined with marital status and farm dimensions, are fundamental elements for technology implementation. Households that have better education and receive stronger extension services tend to adopt conservation agricultural technologies, which enable them to gain benefits (24). Unlike broader studies that generalize adoption patterns, this study reveals that even food-insecure households actively adopt CATs as a strategy to improve resilience and productivity. This insight adds nuance to previous assumptions that only well-resourced or food-secure households engage in agricultural innovation (26, 34). Furthermore, our findings emphasize the need for inclusive policies targeting female-headed households and small-scale farmers, which are often overlooked in mainstream agricultural development programs.

These socioeconomic factors serve as adoption process requirements, yet highlight difficulties in providing equal agricultural technology access to all users. Inclusive policies need to address the gender gap in farming adoption because male farmers outperform female farmers in adopting conservation agricultural technologies. Such technological adoption presents economic limitations that prevent resource-poor households from implementing it; thus, policy reforms must focus on credit access and affordability for wider adoption to succeed (36).

### 4.3 Environmental and economic challenges

All the CATs examined in this research face individual environmental and economic obstacle that may compromise the lasting impact of adoption programs if not properly addressed.

Although intercropping brings the benefits of biodiversity enhancement, improved soil health, and increased sustainability, it faces specific implementation barriers. The literature points to weed management, along with crop competition and complicated resource distribution, as the main challenges that affect mixed cropping systems (37). Smallholder farmers often face difficulties addressing these challenges because they need careful planning and extra management, which exceeds their current capabilities. The widespread adoption of diversified cropping systems faces obstacles because of inconsistent policies as well as the lack of proper support (38). Farmers need better access to specific intercropping management knowledge and proper training, together with the necessary resources, to successfully implement intercropping systems.

Mulching helps the environment by retaining soil moisture, which results in higher crop yields, according to Di Mola et al. (39). Environmental problems emerge from plastic mulch disposal methods, and organic straw mulch enables increased pest attacks (40, 41). The environmental sustainability of mulching materials requires a better understanding, particularly regarding how biodegradable films decrease plastic waste. Biodegradable options need systematic guidelines for use and disposal requirements, and advanced pest control techniques for their successful implementation.

The practice of crop rotation delivers sustainable advantages because it strengthens soil quality while decreasing pesticide requirements (42). Economic factors involving expensive land rents in addition to restricted trade markets of unusual crops create major obstacles for farmers interested in crop diversification (36). Many farmers doubt that crop rotation produces adequate profits since they consider monocropping systems to be more profitable (42). To overcome economic and environmental barriers, the public must become better informed about crop rotation advantages, while governments should establish policies for the market entry of diversified crops.

Soil quality and erosion reduction, along with carbon sequestration, occur through no-tillage farming (43). Initial implementation difficulties of weed control and specialized equipment requirements must be handled through proper management strategies (44). Additional research and environmental monitoring are necessary to prevent long-term environmental deterioration from no-tillage practices, even though they might lead to increased nitrate leaching into groundwater (43). Farmer education about extended advantages and suitable tools and techniques for optimal soil management is essential for sustainable widespread adoption.

### 4.4 Policy implications and the need for awareness

Policy plays an essential dual function by enabling and blocking the implementation of conservation agricultural technologies, according to research findings (45, 46). Applied policy support for intercropping and crop rotation methods produces direct effects on the implementation and maintenance of these farming systems across large areas. This research confirms market access issues, infrastructure shortcomings, and high input expenses as documented challenges across various literature (38). A thorough policy development process must be implemented to minimize the encountered challenges.

According to Banda et al. (47), vital policy frameworks should guide the adoption of conservation agricultural technologies in sub-Saharan Africa. The researchers emphasize that smallholder farmers need policies that unite economics with environmental and social elements to establish an effective framework for their success. The authors of Banda et al. (48) emphasize that agricultural policies need to enhance smallholder farmers’ market access by providing the necessary tools and infrastructure for the successful adoption of new technologies in their farming systems. Government policies should establish financial inclusion by providing low-cost credit together with subsidies to remove financial obstacles that stop farmers from implementing sustainable agricultural practices, as demonstrated by the crop rotation and intercropping system adoption difficulties in this research.

According to Banda et al. (47), agricultural policies should be developed to support sustainability targets. The authors support a policy strategy based on sustainability that tackles both technological adoption programs and fundamental ecological problems in agricultural practices, such as soil deterioration and limited water resources, and environmental consequences. Research data confirms the environmental benefits of using no-tillage and mulching systems, thus demonstrating the vital requirement to manage resources sustainably (49). Hence, Clear guidelines through the incentivized implementation of biodegradable mulches, together with integrated pest management systems, can help policies achieve environmental protection while fully utilizing technological benefits.

Banda et al. (48) emphasize the critical need for inclusive policy development, which ensures all marginalized communities, especially women, obtain equal benefits from CA. Policy solutions must target specific barriers that female agricultural workers encounter because gender-based differences in technology acceptance become obvious from the research findings about adoption metrics. Adequate policies must include gender-sensitive education and improved agricultural service accessibility, along with equal opportunities and resource distribution.

The research results underline the necessity of developing agricultural policies that follow Banda et al. recommendations from 2024a and 2024b. The implementation of agricultural technological adoption policies needs to combine production growth strategies with sustainability practices, which will endure over time. The development of agricultural policies requires extensive consideration of technology adoption’s wider socio-economic and environmental effects so that farmers receive support that handles present and future food security requirements, according to Banda et al. (47). Such an all-encompassing policy framework will assist farmers in choosing appropriate adoption solutions and simultaneously promote sustainable agricultural development across the area.

## 5 Conclusion, limitations and recommendations

Conservation agriculture technologies that include crop rotation, intercropping, mulching, and no-tillage create valuable prospects for better food security and sustainable farming, yet their adoption faces multiple hurdles. The adoption of sustainable technology requires solutions to environmental, economic, and social problems to achieve enduring positive outcomes. The sustainable intensification of agriculture requires farmers to get well-informed about technology complexities while receiving specific policy support and sustainability training to make sustainable choices. Multi-sectoral collaboration between agricultural research services, policy development teams, and market access experts is essential to provide farmers with the tools they need to tackle sustainability problems that emerge during new technology adoption.

The study significantly found the differences between those who adopted and those who did not adopt conservation agricultural technologies in Malawi and uncovered the disparities. Among the disparities, gender is a potential factor in the accessibility of resources and the decision-making process. Furthermore, farm size also proves to be a crucial factor, as the average adopters have higher land per hectare compared to non-adopters. This study uniquely identifies the role of motivational drivers behind CAT adoption among food-insecure households, underscoring the need for targeted interventions such as enhanced extension services, accessible credit facilities for women, and training on complex technologies. These findings offer actionable insights for policymakers aiming to bridge equity gaps while promoting sustainable agricultural practices in similar agro-ecological zones

In addition, farmers with larger household sizes tend to benefit from the contribution of family labor in adopting new practices. Households that adopted technologies experienced lower food insecurity frequencies than non-adopters. Environmental variables such as soil and soil quality, and the type of soil, also influence output; higher temperatures result in poor soil quality, which reduces food production.

This study found that social factors like marital status also play a crucial role in improving food production, as married farmers are more likely to engage in farming activities, unlike single farmers. Therefore, this study recommends educating farmers through training and the best practices to enhance the adoption rate. Thus, investment in agricultural extension programs becomes critical in ensuring that farmers receive the needed training that would enhance the adoption of conservation agricultural technologies. Furthermore, there is also a need to improve access to low-interest credit facilities for female farmers by developing gender-sensitive credit packages specifically targeting female farmers. Moreover, the study highlight the role of social capital such that through information sharing as a family may empower married farmers to engage in CA adoption suggesting that family based farming operations are critical to improve farm management.

The study is not without drawbacks. First, the subjective nature of the data collection instrument predisposes the study findings to recall bias; in order to reduce the bias, the authors pretested the instrument to make sure that it was inclusive. Furthermore, the study was conducted in one district of Malawi; hence, the findings cannot be generalized to the whole country based to some other factors. In addition, the cross-sectional nature of the study makes it impossible to estimate the temporal effects on the variables.

Furthermore, the study recommends an integrated policy approach that promotes sustainability in the adoption of agricultural technology by balancing food security outcomes with economic, social, and environmental factors that influence technology adoption.

## Data Availability

The raw data supporting the conclusions of this article will be made available by the authors, without undue reservation.
