# Supplementary material for: The effect of conservation agriculture technologies adoption on food production and security in Northern Malawi: evidence from Mzimba district
Source: Front Nutr. 2025 Jul 9;12:1615990. doi: 10.3389/fnut.2025.1615990 (PMC12283979; doi:10.3389/fnut.2025.1615990)
Supplement: Supplementary file 1 [file Data_Sheet_1.pdf]

SUPPLEMENTAL FILE

STUDY QUESTIONNAIRE

PART A: SOCIAL DEMOGRAPHIC CHARACTERISTICS

1. What is your Gender
   - Male
   - Female
2. What is your Age-Group
   - 18-25
   - 26-35
   - 35 above
3. Level of Education
   - Primary Level
   - Secondary
   - Tertiary
4. Marital Status
   - Single
   - Married
5. Occupation
   - Crop farming
   - livestock farming
   - trading/business
   - formal employment/civil service
   - piecework
6. Number of Hectors/Acres

   Less than 1 Hector

   2-4 hectors

Above 5 Hectors

7. Type of Farming

Contract

Not on Contract

8. Household Income

Less than MK100,000 per week

MK101-500,000 per week

Above MK500,000 per week

9. Household Size

Less than 2

Above 2 less than 5

Above 5

10. Distance to nearest urban market (IHS, km)

Less than 1km

Above 1km less than 2 km

Above 2km

11. Type of Land ownership

Lease

Rent

Right to farm

PART B: Diversity and Roles of conservation agriculture technologies (CAT) being adopted by rural farmers in Malawi.

12. Have you heard, and have knowledge of CAT

Yes

No

13. Have you adopted CAT

Yes

No

14. If yes, which conservation agricultural technologies (CAT) are you currently using on your farm?

1. Pit Planting/No tillage
2. Mulching
3. Organic Manure
4. Intercropping

15. What are other CAT you know but you are not using, kindly list them.

1. …………………………………
2. …………………………………
3. …………………………………

16. What are the reasons you choose for the CAT you are using?

1. Water conservation
2. Increased crop yield
3. Soil health improvement
4. Reduced labor requirements
5. ……………………………..
6. ……………… ………………

17. In your own understanding are there any specific CAT that are suitable in your EPA (Area, community, village)?

If yes, which one are those?

……

18. Are there any challenges that you think you can face if you want to have more than one CAT in your farm?

If yes, list them?

…….

19. What can you recommend about diversity of CAT.

…….

20. Do you think you have a role to play to increase the diversity of CAT within your area?

…….

PART C: Challenges and opportunities in the adoption of agriculture technologies in Malawi [Adoption patterns]. file:///C:/Users/HP/Downloads/S0305750X21002333.pdf

21. What are the opportunities that you see in adopting CAT? List them

……………………………………………

…………………………………………….

…………………………………………….

…………………………………………..

22. What are the challenges that you face in adopting CAT in your area? List them

………………………………….

…………………………………..

………………………………….

…………………………………..

23. What is the Terrain of your farm
    1. Very steep If the plot is very steep
    2. Moderate slope If the plot moderately sloped
    3. Flat If the plot is flat

PART E: Gender gaps in the adoption of agriculture technologies in Malawi.

24. Gender of the farm manager

Male

Female

25. Status of the farm manager

Single
Married

Widower

Divorced

26. Gender of the one who makes most of the farm decision

Male

Female

Both

27. Is the information on CAT known

Yes

No

28. How do you rate time while working on CAT

Time consuming

Moderate

Not time consuming

29. How do you rate complexity while working on CAT

Not complex

Moderate

More complex

30. Do you think CAT helps in Value creation of crops per acres.

Yes

No

31. Do you think CAT helps in quantity of crops per acres.

Yes

No

PART F: Adoption factors of CAT in Malawi.

32. Extension access

Yes

No

33. Farming experience

Less than 5 Years

Above 5 years

34. Ownership of a smartphone and ownership of a radio

Yes

No

35. Belonging to Club or Association

Yes

No

36. Training CAT Attainment

Yes

No

37. Soil type

Sandayloam

Loam

Clay

38. Temperature

Hot

Cold

39. Rainfall

Less …mm

Above …mmm

40. Soil Quality

Good

Average

Poor

41. Reasons for CAT adoption-components using a Likert scale of SD-SA

    a. Technicality
    b. Financial
    c. Awareness
    d. Infrastructure
    e. Social influence

PART G: Effects of agriculture technologies adoptions on Food production and Security in Malawi.

SD questions

42. Economical

43. Social

44. Environmental

45. FOODSEC-Food Production and Food security Household Consumption Score.

Please answer whether this happened and frequency of occurrence on scale, rarely (once or twice), sometimes (3 -10 times) or often (more than 10 times) in the past four weeks. [Food – staple food, animal, fruits, vegetables etc]

No. HFIAS Occurrence: 1=Yes: 0=No Frequency-of-occurrence: How often did this happen in the past four weeks? 1 = Rarely (once or twice) 2 = Sometimes (three to ten times) 3 = Often (more than ten times)

Questions

a. In the past four weeks, did you worry that your household would not have enough food?

b. In the past four weeks, were you or any household member not able to eat the kinds of foods you preferred because of a lack of resources?

c. In the past four weeks, did you or any household member have to eat a limited variety of foods due to a lack of resources?

d. In the past four weeks, did you or any household member have to eat some foods that you really did not want to eat because of a lack of resources to obtain other types of Food?

e. In the past four weeks, did you or any household member have to eat a smaller meal than you felt you needed because there was not enough food?

f. In the past four weeks, did you or any household member have to eat fewer meals in a day because there was not enough food?

g. In the past four weeks, was there ever no food to eat of any kind in your household because of lack of resources to get food?

h. In the past four weeks, did you or any household member go to sleep at night hungry because there was not enough food?

i. In the past four weeks, did you or any household member go a whole day and night without eating anything because there was not enough food?

END OF THE QUESTIONNAIRE
